# Supplementary material for: Intranasal Immunization with Live-Attenuated RSV-Vectored SARS-CoV-2 Vaccines Elicits Antigen-Specific Systemic and Mucosal Immunity and Protects Against Viral Challenge and Natural Infection
Source: Vaccines (Basel). 2026 Apr 29;14(5):399. doi: 10.3390/vaccines14050399 (PMC13211619; doi:10.3390/vaccines14050399)
Supplement: Supplementary file 1 [file vaccines-14-00399-s001.zip › vaccines-4248451-supplementary.pdf]

Supplemental Figures and Legends

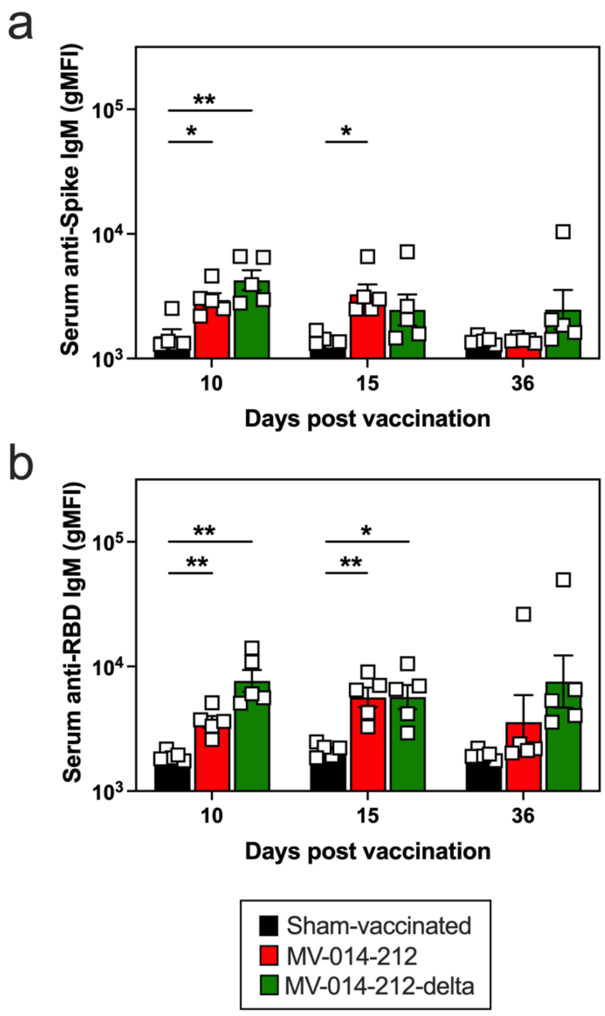

Figure S1. Systemic antigen-specific IgM responses in mice following a single i.n. administration of MV-014-212 or MV-014-212-delta vaccine. Spike-specific IgM (**a**) and RBD-specific IgM (**b**) in sera from K18-hACE2 mice at days 10, 15 and 36 post vaccination with Vero cell lysate (sham vaccine; black), MV-014-212 (red) or MV-014-212-delta (green). Data plotted as log-transformed geometric mean fluorescence intensity (gMFI). Individual animals (n=5/group) shown as symbols and means of groups shown as bars. *P <0.05, **P<0.01. P values determined by one-way ANOVA followed by Dunnett's multiple comparison test. Variability expressed as standard error (SE).

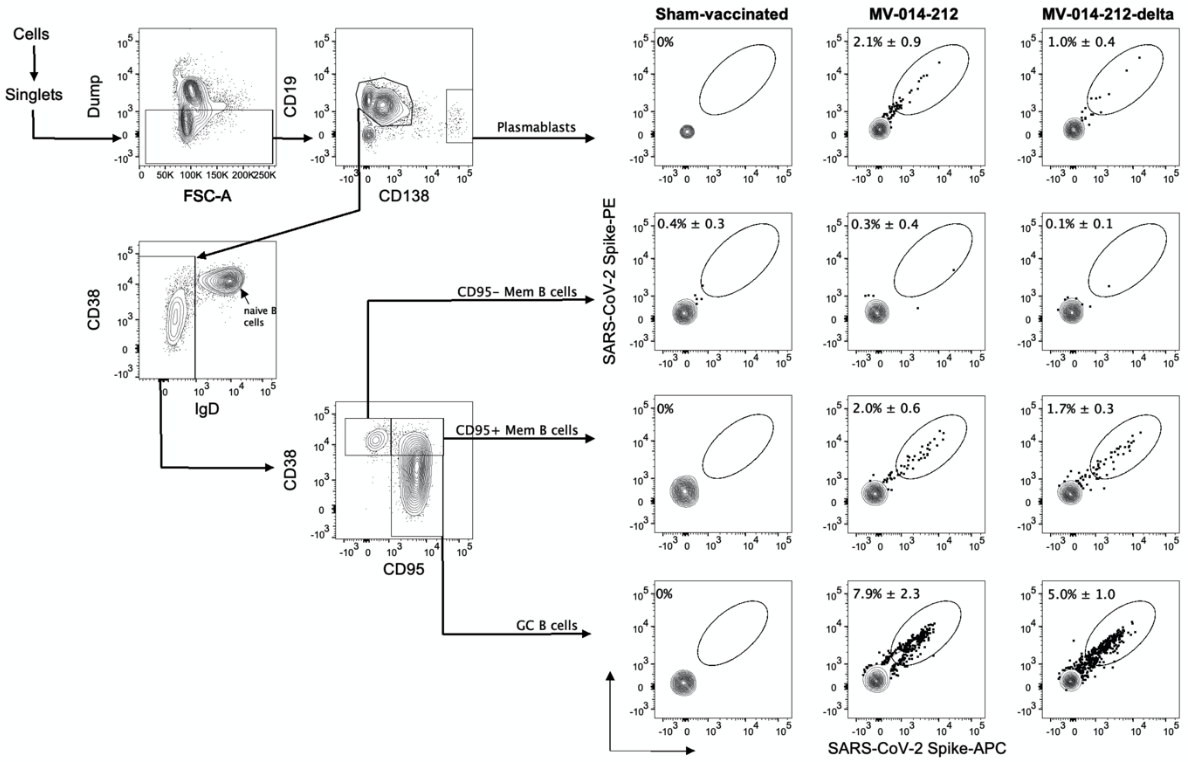

Figure S2. Gating strategy for flow cytometric analysis of Spike-specific B cells in the mediastinal lymph node from vaccinated mice. Sequential gating strategy for identification and enumeration of Spike-specific plasmablasts, $CD95^+$ memory (Mem) B cells, $CD95^{neg}$ Mem B cells and germinal center (GC) B cells in the mediastinal lymph node of K18-hACE2 mice at day 10 post vaccination with Vero cell lysate (sham-vaccinated), MV-014-212 or MV-014-212-delta. A dump channel containing 7-Aminoactinomycin D (7-AAD) viability dye, anti-mouse F4/80 and anti-mouse CD3 antibodies was used to exclude dead cells, macrophages and T cell lymphocytes from the analysis. Plasmablasts were phenotyped as $Dump^{neg}$ $CD19^{+/lo}$ $CD138^+$ cells. Mem B cells were phenotyped as $Dump^{neg}$ $CD19^+$ IgD^{neg} $CD38^{hi}$ cells. Mem B cells were further subdivided into $CD95^{neg}$ and $CD95^+$ Mem B cell subsets. GC B cells were phenotyped as $Dump^{neg}$ $CD19^+$ IgD^{neg} $CD38^{neg}$ $CD95^+$ cells. Spike-specific B cell populations were identified as binding to recombinant SARS-CoV-2 Spike tetramers conjugated to PE or APC. The frequency of B cells dual stained with the two Spike tetramers is expressed as percentage ± standard deviation (STDEV).

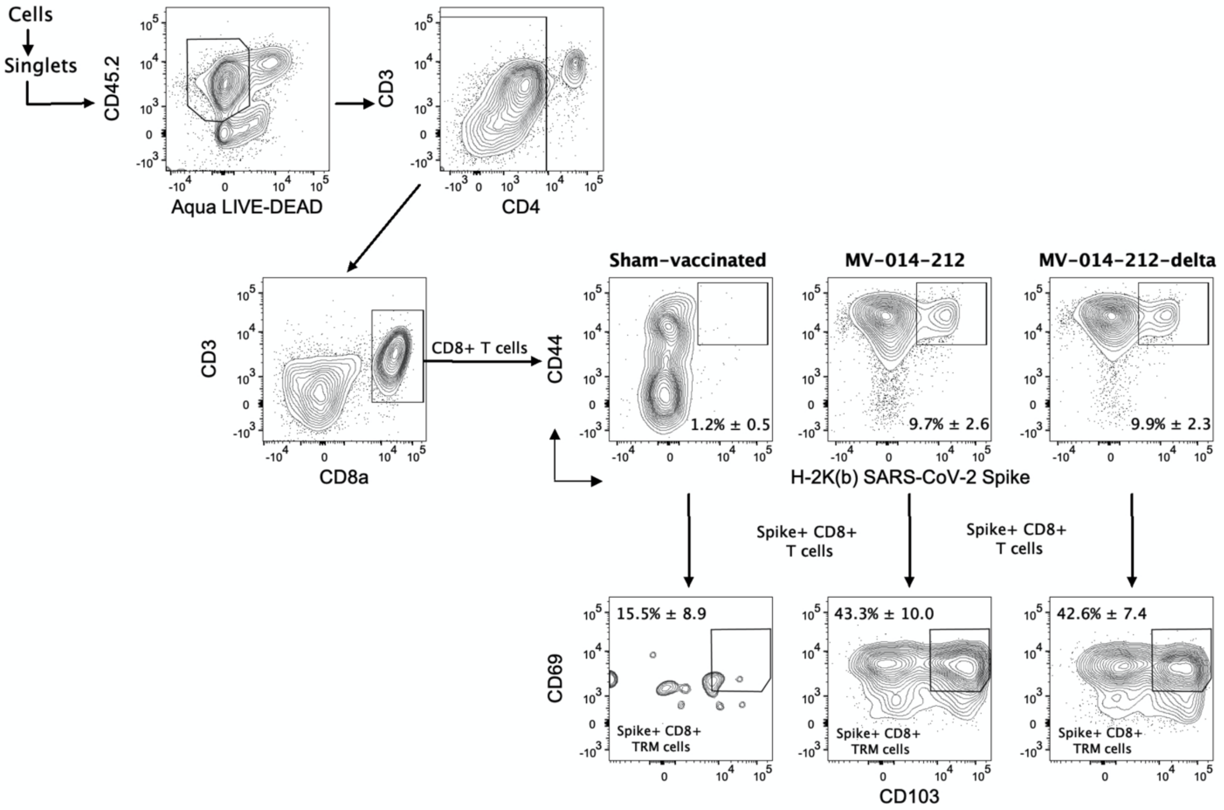

Figure S3. Gating strategy for flow cytometric analysis of Spike-specific CD8$^+$ T cells in the lungs from vaccinated mice. Sequential gating strategy for the identification and enumeration of total Spike-specific CD8$^+$ T cells and Spike-specific tissue-resident memory (TRM) CD8$^+$ T cells in the lungs of K18-hACE2 mice at day 10 post vaccination with Vero cell lysate (sham-vaccinated), MV-014-212 or MV-014-212-delta. CD45.2$^+$ live (Aqua^{neg}) cells were gated and total CD8$^+$ T cells were identified as CD4^{neg} CD3$^+$ CD8$^+$ cells. Spike-specific CD8$^+$ T cells were identified with fluorochrome-labeled Class I MHC H-2K^b SARS-CoV-2 Spike (VNFNFNGL)-BV421 tetramers. Spike-specific CD8$^+$ TRM cells were identified based on dual expression of CD69$^+$ and CD103$^+$. The frequency of each Spike-specific CD8$^+$ T cell population is expressed as percentage ± STDEV.

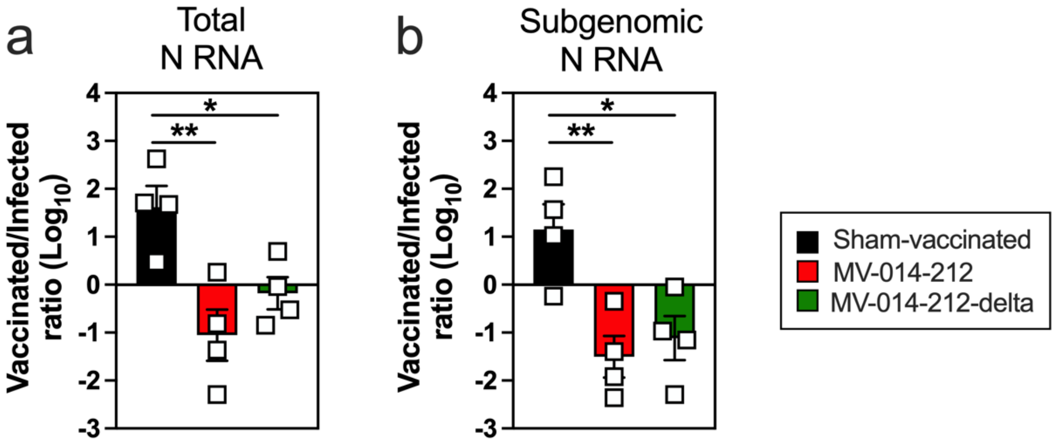

Figure S4. Viral N transcripts in nasal swipe samples collected from infected animals and vaccinated cagemates. Comparison of total (**a**) and subgenomic (**b**) viral N RNA levels from nasal swipes collected from experimentally-infected hamsters and their vaccinated (red and green) or sham-vaccinated (black) cagemates. Data are reported as the ratio of viral N RNA transcript levels (copies/ml) in nasal swipes from day 5 Delta SARS-CoV-2-infected animals to those collected from vaccinated/sham-vaccinated cagemates at day 5 post pairing. Animal pairs (n=4/group) are shown as symbols with the means of the ratios reported as bars. Data plotted as log-transformed ratios. *P <0.05, **P<0.01. P values determined by one-way ANOVA followed by Dunnett's multiple comparison test. Variability expressed as SE.
